# The Effects of Vibration Therapy on Activities of Daily Living After Stroke: A Systematic Review and Meta-Analysis

**DOI:** 10.3390/jcm14217682

**Published:** 2025-10-29

**Authors:** Jeong-Woo Seo, Jaeuk. U. Kim, Jung-Dae Kim, Ji-Woo Seok

**Affiliations:** Digital Health Research Division, Korea Institute of Oriental Medicine, 1672 Yuseong-daero, Yuseong-gu, Daejeon 34054, Republic of Korea; jwseo02@kiom.re.kr (J.-W.S.);

**Keywords:** activities of daily living, vibration therapy, stroke, whole-body vibration, focal muscle vibration

## Abstract

**Background/Objectives**: Activities of daily living (ADL) are critical for independence after stroke, yet many survivors remain functionally limited. Vibration therapy (VT), including whole-body and focal modalities, has been proposed as an adjunct to enhance recovery, but effects on ADL remain unclear. This study aimed to evaluate the overall effectiveness of VT on ADL and to identify moderating factors. **Methods**: A systematic review and meta-analysis were conducted following PRISMA 2020 guidelines. Thirteen controlled trials (12 RCTs, 1 nRCT) involving VT in stroke were included. Standardized mean differences (Hedges’ g) were synthesized using random-effects models. Meta-regression and subgroup analyses examined moderators such as session number, vibration parameters, stroke stage, and ADL subdomains. Risk of bias was assessed with RoB 2 and ROBINS-I. **Results**: VT produced a small but significant effect on ADL (Hedges’ g = 0.19; 95% CI: 0.06–0.33; *p* = 0.008), though significance was lost after adjustment for publication bias. Heterogeneity was moderate (I^2^ = 34%). Session number was the only significant moderator (*p* = 0.045), explaining ~24% of variance, with the greatest benefit in the 13–24 session range (g = 0.34; 95% CI: 0.05–0.63). Subgroup analysis showed improvement in physical function/mobility (g = 0.32; *p* = 0.048), but not in self-care or quality-of-life outcomes. Other parameters were not significant moderators. **Conclusions**: VT confers modest benefits for ADL after stroke, particularly in mobility-related domains. Session number appears clinically important, with 13–24 sessions suggesting an optimal dose window.

## 1. Introduction

Stroke remains one of the leading causes of long-term disability worldwide, often resulting in persistent impairments in motor function, balance, sensory control, and, critically, the ability to perform activities of daily living (ADL). ADL refers to the basic and instrumental tasks necessary for independent living and is typically divided into basic ADL (e.g., feeding, dressing, bathing, toileting, mobility) and instrumental ADL (e.g., cooking, shopping, managing finances, using transportation) [1]. Post-stroke ADL limitations are closely linked to neurological deficits, including upper and lower limb motor impairment, balance dysfunction, gait abnormalities, cognitive decline, and language disorders. For example, hemiplegic patients often experience severe difficulties in basic ADL due to upper-limb fine motor deficits, while weakness and impaired proprioception in the lower limbs delay gait recovery and independence [2,3]. Moreover, cognitive and language impairments, such as aphasia and executive dysfunction, restrict the ability to perform instrumental ADL tasks requiring planning, problem-solving, and social interaction, leading to long-term functional dependency [4].

To objectively quantify ADL performance, several standardized assessment tools have been developed. The Barthel Index (BI) and its modified version (MBI) remain the most widely used instruments for evaluating basic ADL, with lower scores indicating greater dependency [5]. The Functional Independence Measure (FIM) incorporates both physical and cognitive domains for a more comprehensive assessment of functional recovery [6]. The Lawton Instrumental Activities of Daily Living (IADL) scale assesses higher-order instrumental tasks, such as managing finances, transportation, and household responsibilities [7]. These instruments provide essential benchmarks for monitoring functional recovery trajectories and establishing rehabilitation goals in stroke populations.

Clinically, longitudinal studies have reported that stroke patients dependent in ADL within the first 36–48 h post-onset are likely to remain dependent at 3 and 12 months [8]. Moreover, more than 20% of stroke survivors exhibit persistent limitations in basic ADL, and over 30% experience restrictions in instrumental ADL [9]. These limitations are associated not only with physical disability but also with reduced quality of life, depression, increased fall risk, and higher rates of institutionalization [10,11]. Therefore, ADL recovery represents a core objective in stroke rehabilitation, encompassing both physical independence and psychosocial well-being.

Recently, vibration therapy has emerged as a promising adjunctive intervention in neurorehabilitation. Two main modalities have been described: whole-body vibration (WBV), delivered via oscillating or side-alternating platforms, and focal muscle vibration (FMV), applied locally to specific muscle groups or tendons [12,13]. Physiologically, vibration stimuli activate muscle spindles and Ia afferent fibers, eliciting the tonic vibration reflex, which can enhance muscle activation, proprioceptive input, and neuroplasticity, thereby facilitating motor recovery and postural control [14,15].

However, evidence regarding the effects of vibration therapy on ADL improvement remains inconclusive. Previous meta-analyses have primarily focused on motor function, gait, or balance outcomes [16], while effect heterogeneity, dose–response relationships, and domain-specific effects have not been systematically examined. In particular, uncertainties persist regarding whether intervention parameters—such as total number of sessions, amplitude, frequency, or duration—serve as moderators influencing treatment efficacy. Moreover, it remains unclear whether different ADL domains (e.g., self-care/daily activities, physical function/mobility, and quality of life/participation) respond differently to vibration therapy, limiting the development of targeted rehabilitation strategies.

To address these gaps, this study conducted a systematic review and meta-analysis to quantify the overall effect size of vibration therapy on ADL improvement in stroke patients, while simultaneously evaluating effect heterogeneity and identifying potential moderators such as total session number through meta-regression analyses. Furthermore, we explored domain-specific effects across ADL subcategories to determine not only whether vibration therapy improves ADL but also under what conditions and in which functional domains its effects are most pronounced, thereby providing evidence-based recommendations for optimizing vibration therapy protocols in stroke rehabilitation, particularly regarding the optimal dose window and target outcomes for clinical practice.

## 2. Methods

### 2.1. Study Design

This study was conducted as a systematic review and meta-analysis to comprehensively evaluate the effects of vibration therapy on improving ADL in stroke patients. The study adhered to the PRISMA 2020 guidelines (Table S1) [17], and the protocol was registered in PROSPERO (registration number: CRD420251110158).

### 2.2. Literature Search and Screening

A systematic literature search was conducted according to a predefined protocol. The primary databases searched were CENTRAL (Cochrane Central Register of Controlled Trials), Embase (via Ovid), MEDLINE, PubMed, and the Science Citation Index (SCI), with no restrictions on publication year or language.

The search strategy was developed around key concepts, including stroke, vibration therapy, and ADL, and incorporated both Medical Subject Headings (MeSH) terms and free-text keywords. Additional studies were identified by screening reference lists and contacting experts in relevant fields.

All retrieved articles were screened in a stepwise process by two independent reviewers based on predefined inclusion and exclusion criteria. Titles and abstracts were first assessed to identify potentially relevant studies, followed by full-text reviews for final inclusion. Any disagreements between the reviewers were resolved through consultation with a third reviewer.

### 2.3. Eligibility Criteria

This study included adults aged 18 years or older diagnosed with ischemic or hemorrhagic stroke, who reported impairments in ADL across all stages of recovery (acute, subacute, and chronic). Eligible interventions were whole-body vibration (WBV) or focal muscle vibration (FMV) with clearly defined parameters, administered either alone or in combination with conventional rehabilitation.

Control conditions comprised no treatment, sham vibration (e.g., standing on a platform without vibration), general exercise, or cognitive training. Studies in which WBV was combined with another intervention were also included if the control group received the same intervention alone (e.g., experimental group = WBV + exercise, control group = exercise), allowing for the estimation of the pure effects of WBV. Conversely, studies in which WBV was combined with another intervention, but the control group received no treatment or sham vibration alone, were excluded, as the effects of WBV could not be separated from those of the additional intervention.

Primary outcomes were restricted to quantitative ADL-related assessment tools, including the Barthel Index (BI or modified BI), Functional Independence Measure (FIM), CHIEF-C, Frenchay Activities Index (FAI), the ADL domain of the Stroke-Specific Quality of Life (SS-QOL), and the SF-12 Physical Composite Score (PCS). Eligible study designs included randomized controlled trials (RCTs) and non-randomized controlled trials (nRCTs). Studies were excluded if they investigated populations other than adults with stroke (e.g., children), were preclinical or cell/animal experiments, did not report ADL-related outcomes, lacked sufficient statistical data (e.g., means, standard deviations, or *p*-values), involved passive vibration or combined interventions in which the effects of WBV could not be isolated, or were case reports, case series, cross-sectional studies, observational cohort studies, case–control studies, narrative reviews, editorials, or conference abstracts.

### 2.4. Data Extraction

Data extraction was independently performed by two researchers using a pre-established standardized form. Information collected included author names, publication year, study design, participant characteristics (age, sample size, and clinical stage), intervention and control protocols, and the type of equipment used. To characterize vibration interventions in detail, data were extracted according to the “WBV Big Five” core variables described in previous research [18], namely frequency, amplitude, application method, session duration, session frequency, and total intervention period. Primary outcome measures were restricted to standardized assessment tools of ADL, encompassing subdomains of self-care and daily activities, quality of life and participation, and physical function and mobility.

For studies presenting numerical data only in graphical form, values were digitized using WebPlotDigitizer (ver. 4). When statistical parameters such as means and standard deviations were not directly available, additional data were requested from study authors via email. If only medians or quartiles were reported, means and standard deviations were estimated using established statistical methods. Any discrepancies during the extraction process were resolved through consensus.

### 2.5. Assessment of Methodological Quality

Included randomized controlled trials (RCTs) were evaluated using the Cochrane Risk of Bias 2 (RoB 2) tool, which assesses potential bias across domains such as the randomization process, deviations from intended interventions, missing outcome data, outcome measurement, and selective reporting [19].

Non-randomized controlled trials (nRCTs) were evaluated with the ROBINS-I (Risk of Bias in Non-randomized Studies of Interventions) tool, covering seven domains: confounding, selection of participants, classification of interventions, deviations from intended interventions, missing data, measurement of outcomes, and selection of the reported result [20]. All assessments were conducted independently by two reviewers, and disagreements were resolved through discussion and consensus.

### 2.6. Statistical Analysis

All statistical analyses were conducted using the metafor package in the R statistical software environment. Effect sizes were calculated as Hedges’ g based on the reported results of each study, following the recommendations of the Cochrane Handbook [21,22]. For studies reporting mean changes and standard deviations from pre- to post-treatment, these values were directly used to calculate between-group differences. When only pre- and post-treatment means and SDs were reported, the SD of the change was estimated using an assumed correlation coefficient of 0.5, after which Hedges’ g was calculated [22,23,24].

Pooled effect sizes were synthesized using a random-effects model, with the restricted maximum likelihood (REML) estimation methods applied to account for between-study variability and to ensure more conservative estimates. Heterogeneity was evaluated using the chi-square test (Q statistic) and the I^2^ statistic, with thresholds of 25%, 50%, and 75% interpreted as low, moderate, and high heterogeneity, respectively. In addition, τ^2^ (between-study variance) and the H^2^ index were calculated to provide a comprehensive assessment of heterogeneity [22,25]. Predictive intervals were also reported to indicate the expected range of effects in future studies.

Publication bias was assessed by visual inspection of funnel plots as well as Egger’s regression test and Begg’s test [26,27,28]. Where asymmetry was suspected, the trim-and-fill method was applied to estimate adjusted effect sizes. A fail-safe N was also computed to evaluate the robustness of the findings [29].

Meta-regression analyses were performed to explore potential sources of heterogeneity. Moderator variables included characteristics of the vibration intervention (e.g., vibration type, frequency category, amplitude level, session duration, and total number of sessions) and study-level factors (e.g., clinical stage and outcome domain). The significance of moderator effects was tested using the Knapp–Hartung adjustment [30].

Finally, subgroup analyses were conducted based on the subdomains of ADL assessment tools, categorized as (1) self-care and daily activities, (2) physical function and mobility, and (3) quality of life and participation. Effect sizes and heterogeneity were calculated separately within each subgroup, and differences between subgroups were tested using Cochran’s Q test for subgroup differences (Qm test) [31].

## 3. Results

### 3.1. Study Selection

**Figure 1:**
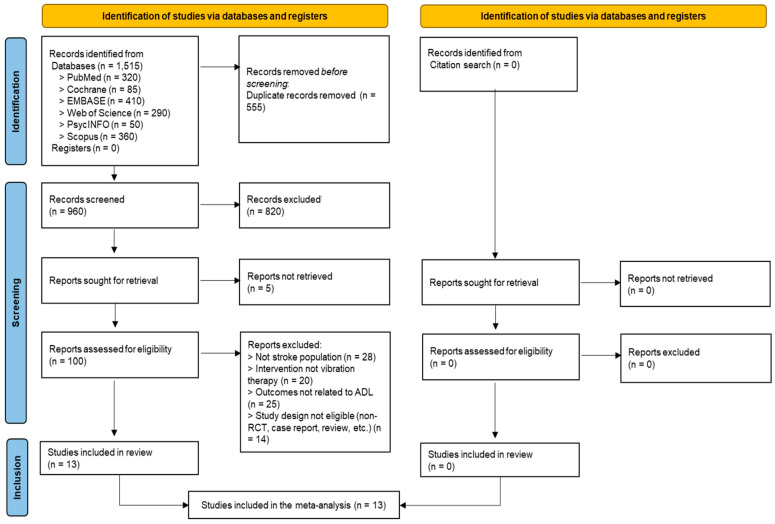
Flowchart of study selection.

A total of 1515 records were initially identified through database searches. After the removal of 555 duplicates, 960 records remained for title and abstract screening. Of these, 820 records were excluded, leaving 140 articles for full-text review. Five articles were excluded due to unavailable full-texts, and 95 were assessed for eligibility. Among these, 28 studies were excluded for not involving stroke patients, 20 for not using vibration therapy, and 25 for not reporting ADL-related outcomes. In addition, 14 studies were excluded due to ineligible study designs (e.g., non-randomized trials, case reports, or reviews). Ultimately, 13 studies were included in this systematic review and meta-analysis. The study selection process is illustrated in the PRISMA flow diagram (Figure 1).

### 3.2. Characteristics of the Studies

The 13 included studies were published between 2006 and 2025. Of these, 12 were RCTs and one was a non-RCT (Table 1). Seven studies targeted patients in the subacute phase, while six focused on patients in the chronic phase. Sample sizes ranged from 9 to 64 participants, with mean ages generally spanning from the late 50s to early 70s. Nine studies employed WBV, and four applied focal muscle vibration (FMV). WBV interventions primarily used frequencies ranging from 16 to 40 Hz and amplitudes of 1–4 mm, delivered in various postures such as squatting, sitting, and standing. FMV interventions applied frequencies of 30–300 Hz, typically localized to upper limb muscles or lower limb tendons. The intervention duration ranged from 2 to 8 weeks, with session frequencies ranging from 3 to 10 per week. Devices included commercially available platforms and localized vibration instruments such as Galileo^®^ (Novotec Medical GmbH, Pforzheim, Germany), Vibrosphere (ProMedVi, Lund, Sweden), Sonix (Sonic Life, Hood River, OR, USA), Gymna Fitvibe (Gymna-Uniphy, Bilzen, Belgium), and Viss^®^ (Vissman, Rome, Italy).

All studies utilized standardized assessment tools related to ADL, which were categorized into three subdomains: (1) The self-care and daily activities domain included the Barthel Index (BI), Frenchay Activity Index (FAI), Functional Independence Measure (FIM), the Korean version of the Modified Barthel Index (K-MBI), and the Modified Barthel Index (MBI). (2) The physical function and mobility domain included the Trunk Control Test (TCT), Rivermead Mobility Index (RMI), Quick Disabilities of the Arm, Shoulder, and Hand Questionnaire (QDS), Functional Ambulation Categories (FAC), Trunk Impairment Scale (TIS), and Modified Postural Assessment Scale for Stroke (MPASS). (3) The quality of life and participation domain included the Craig Hospital Inventory of Environmental Factors–Chinese version (CHIEF-C), the 12-Item Short Form Health Survey (SF-12; PCS, MCS), and the Stroke-Specific Quality of Life (SS-QOL) (Table 1). Across all included studies, no adverse events such as dizziness, musculoskeletal discomfort, or falls were reported in either the vibration or control groups.

### 3.3. Quality Assessment Results

**Figure 2:**
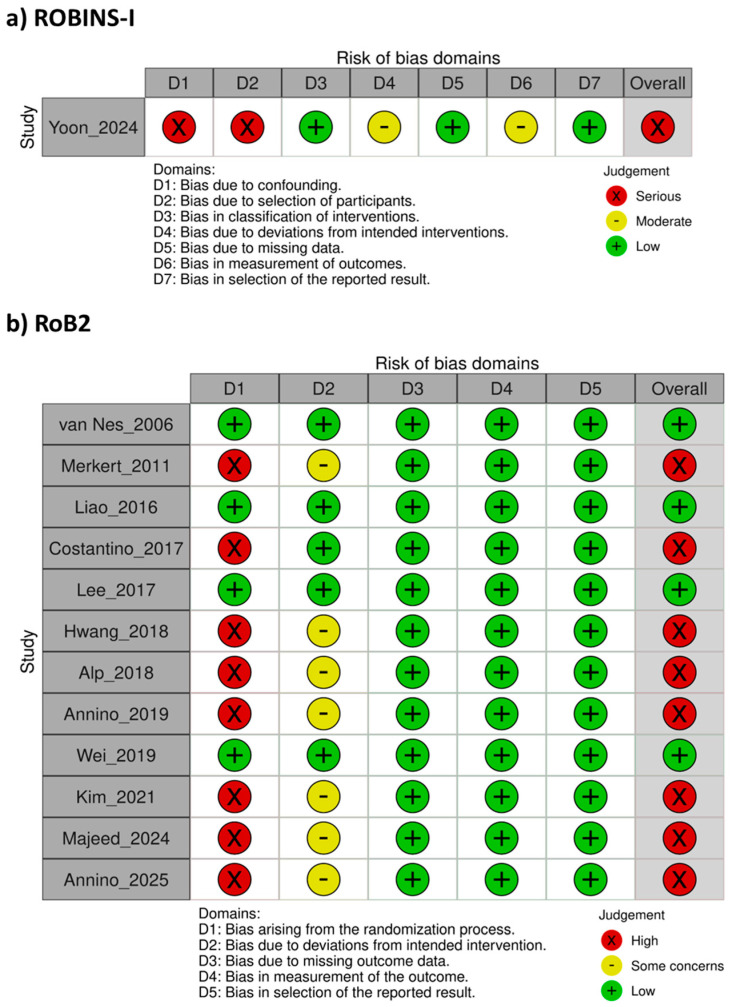
Methodological quality assessment of the included studies with the Cochrane scale [32,33,34,35,36,37,38,39,40,41,42,43,44].

One nRCT was evaluated using the ROBINS-I tool and was judged to have a serious overall risk of bias, primarily due to confounding and participant selection (Figure 2).

Twelve RCTs were assessed using the Cochrane Risk of Bias 2 (RoB 2) tool. In the randomization process domain, eight studies (66.7%) were judged to have a high risk of bias owing to unclear sequence generation or lack of reporting on allocation concealment, whereas four studies (33.3%) were considered low risk. With respect to deviations from intended interventions, five studies (41.7%) were assessed as low risk due to faithful adherence to the planned protocol, while seven studies (58.3%) raised some concerns because of the absence of participant or investigator blinding or the failure to perform intention-to-treat analyses. All studies were rated low risk in the domains of missing outcome data, outcome measurement, and selective reporting, indicating adequate data handling and outcome reporting.

Overall, four RCTs (33.3%) were judged to have an overall low risk of bias, whereas eight (66.7%) were rated as high risk, suggesting that methodological limitations were most evident in randomization and intervention management procedures (Figure 2).

### 3.4. Effect of Whole Body Vibration on ADL

**Figure 3:**
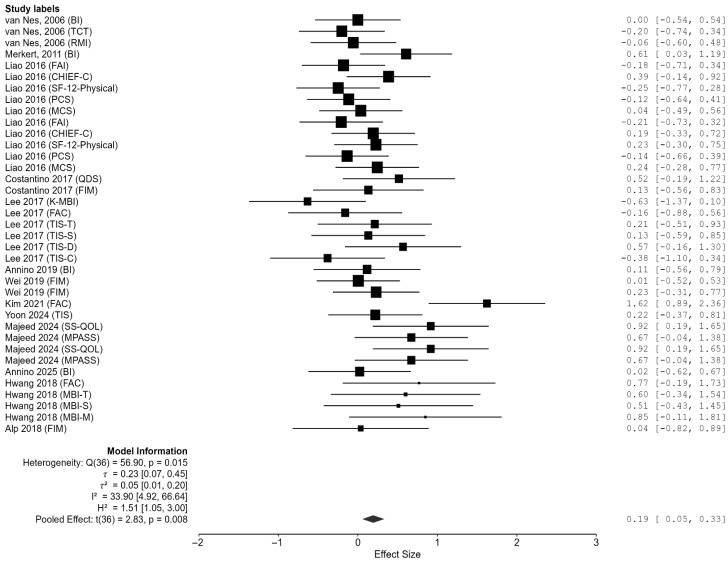
Meta-analysis of the effects of WBV on ADL [32,33,34,35,36,37,38,39,40,41,42,43,44].

This meta-analysis included 13 RCTs evaluating the effects of WBV on ADL in stroke patients (Figure 3). The pooled effect size was Hedges’ g = 0.194 (95% CI = 0.055–0.333, *p* = 0.008), indicating that WBV produced a small but statistically significant improvement in ADL compared with control conditions (Figure 3).

The test for heterogeneity was significant (Q(36) = 56.90, *p* = 0.015), with I^2^ = 33.9% (95% CI = 4.9–66.6%), reflecting moderate between-study variability. Additional indices also indicated notable heterogeneity (τ = 0.228, 95% CI = 0.073–0.451; τ^2^ = 0.052, 95% CI = 0.005–0.203; H^2^ = 1.513, 95% CI = 1.052–2.998). These findings suggest that differences in study design, participant characteristics, and outcome assessment methods may account for the observed variability.

**Figure 4:**
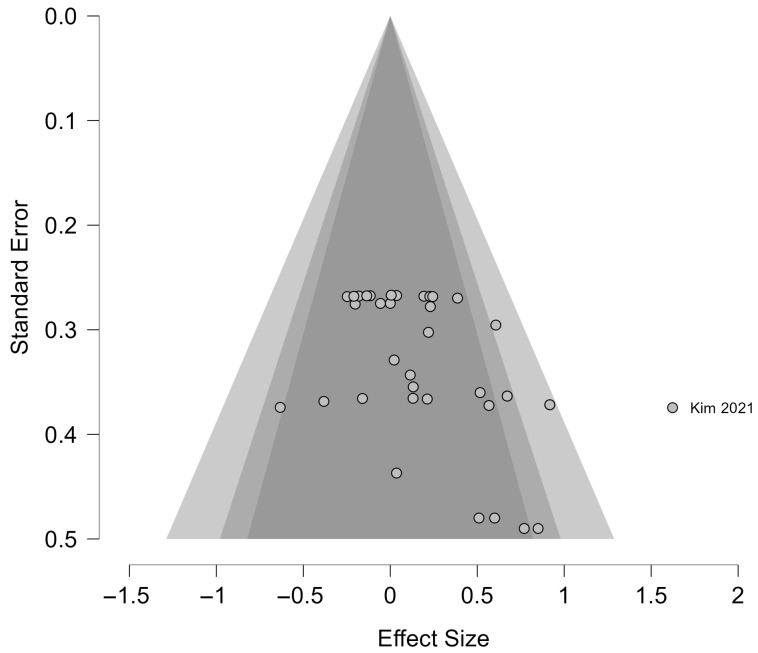
Funnel plot assessing publication bias in the meta-analysis of WBV effects on ADL [39].

Publication bias was examined using funnel plots and Egger’s regression test (Figure 4). Egger’s test revealed significant asymmetry (z = 3.294, *p* < 0.001), raising concerns about publication bias. After adjustment with the trim-and-fill method, however, the pooled effect was no longer statistically significant (g = 0.074, 95% CI = −0.079–0.227, *p* = 0.343). Rosenthal’s Fail-Safe N indicated that 169 unpublished studies with null results would be required to overturn the observed effect, providing some reassurance regarding the robustness of the findings.

In summary, WBV was associated with a small but significant improvement in ADL among stroke patients. Nonetheless, this effect was accompanied by moderate heterogeneity and potential publication bias, and the adjusted estimate did not reach significance. These results underscore the need for further validation in large-scale, methodologically rigorous trials.

### 3.5. Meta-Regression of Moderators of WBV Effects on ADL

In this meta-regression analysis, we evaluated several moderator variables that could influence the effects of WBV on ADL in stroke patients (Table 2). Stroke type (F(1, 35) = 0.061, *p* = 0.807), vibration type (F(1, 35) = 0.053, *p* = 0.820), frequency type (F(3, 33) = 0.378, *p* = 0.769), amplitude type (F(1, 22) = 1.430, *p* = 0.245), time group (F(2, 32) = 0.857, *p* = 0.434), and outcome domain (F(2, 34) = 0.762, *p* = 0.474) were not significant moderators. By contrast, the session group was identified as a significant moderator (F(2, 34) = 3.41, *p* = 0.045). Participants receiving 13–24 sessions demonstrated significantly greater improvements (g = 0.34, 95% CI = 0.048–0.632, *p* = 0.024) compared with those receiving ≥25 sessions, whereas ≤12 sessions showed no significant effect (g = 0.005, 95% CI = −0.356–0.367, *p* = 0.976). These findings suggest that a moderate number of repeated interventions may be particularly beneficial for ADL improvement.

The influence of moderators on heterogeneity was also examined (Table 3). In the baseline model, τ^2^ was 0.052, and I^2^ was 33.9%. Stroke type, vibration type, time group, frequency group, and outcome domain did not meaningfully reduce residual heterogeneity. Session group, however, significantly moderated the effects (Q(34) = 48.85, *p* = 0.048), lowering τ^2^ to 0.044 and thereby partially accounting for between-study variability. For the amplitude group, residual heterogeneity was estimated as zero, likely reflecting model instability due to reduced sample size.

### 3.6. Sensitivity Analysis

To test the robustness of the findings, a sensitivity analysis was performed after excluding three types of studies: (1) influential outliers identified by DFFITS and Cook’s distance (Kim 2021, [FAC] [39] and Lee 2017, [K-MBI] [36]) and (2) non-randomized trial (Yoon et al., 2023, [40]).

The outliers were excluded because Kim (2021) [39] exhibited the highest DFFITS and Cook’s distance values, indicating high influence on the pooled estimate, while Lee (2017) [36] had a standardized residual of −1.87, which is close to the conventional cutoff value (±1.96). Non-randomized studies were removed to assess whether study design heterogeneity influenced the results. After excluding these studies, the overall effect remained significant and stable (Hedges’ g = 0.16, 95% CI [0.04, 0.28], *p* = 0.009), with no significant heterogeneity (Q(33) = 37.16, *p* = 0.283; τ^2^ = 0.006). The 95% prediction interval ranged from −0.03 to 0.35, indicating that most true effects across comparable populations are expected to be positive. These results confirm that the overall conclusion is robust and not driven by outliers or non-randomized studies.

### 3.7. Subgroup Analysis of WBV Effects Across ADL Domains

**Figure 5:**
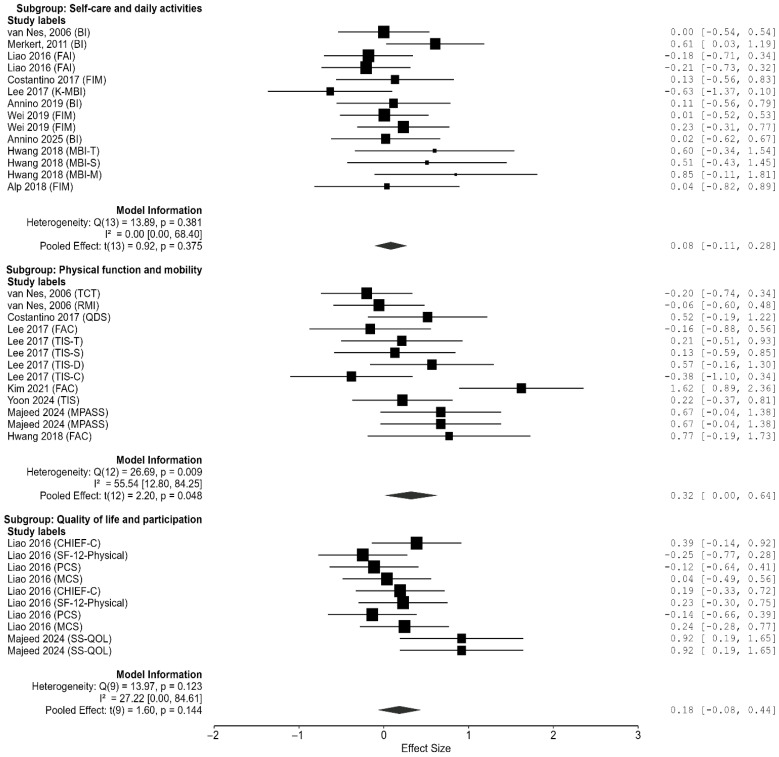
Forest plots showing the effects of WBV on three ADL domains [32,33,34,35,36,37,38,39,40,41,42,43,44].

**Figure 6:**
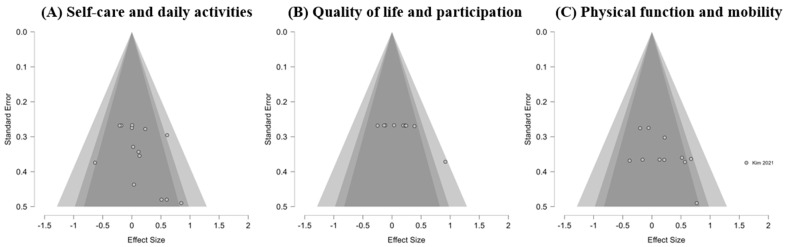
Funnel plots for the assessment of publication bias in three ADL domains [39].

A subgroup analysis was conducted to evaluate the effects of WBV interventions on ADL across three domains: self-care and daily activities, physical function and mobility, and quality of life and participation (Figure 5 and Figure 6).

For the self-care and daily activities domain, the pooled effect size was not significant (g = 0.083, 95% CI [−0.112, 0.279], *p* = 0.375), with low heterogeneity (Q_e_(13) = 13.89, *p* = 0.381, I^2^ ≈ 0%). In the physical function and mobility domain, a significant positive effect was detected (g = 0.323, 95% CI [0.003, 0.642], *p* = 0.048). However, heterogeneity was moderate to high (Q_e_(12) = 26.69, *p* = 0.009, I^2^ ≈ 55.5%). For the quality of life and participation domain, the pooled effect size was again non-significant (g = 0.184, 95% CI [−0.076, 0.445], *p* = 0.144), with low heterogeneity (Q_e_(9) = 13.97, *p* = 0.123, I^2^ ≈ 27.2%).

A test for subgroup differences indicated no significant variation in effect sizes across the three domains (Q_m_(2) = 2.00, *p* = 0.369). Overall, WBV did not exert consistent effects across ADL subdomains, with statistically significant improvements observed only in physical function and mobility.

Effect sizes (Hedges’ g) and 95% confidence intervals are displayed for each study across the following domains: (A) self-care and daily activities, (B) quality of life and participation, and (C) physical function and mobility. Pooled estimates were calculated using a random-effects model.

Each panel presents a funnel plot of standard error against effect size for studies evaluating (A) self-care and daily activities, (B) quality of life and participation, and (C) physical function and mobility.

## 4. Discussion

This systematic review and meta-analysis aimed to quantify the effects of vibration therapy on activities of daily living (ADL) improvement in stroke patients, explore sources of heterogeneity, and identify clinically meaningful prescription parameters. Overall, the pooled effect was small but statistically significant; however, statistical significance was lost after sensitivity analyses, including publication bias adjustment. Moderate heterogeneity was observed, indicating the need to investigate moderators. Meta-regression revealed that only the total number of sessions was a significant moderator, with the most pronounced effects observed in the 13–24 session range. In addition, subgroup analysis indicated significant improvements only in the physical function/mobility domain, but not in self-care/daily activities or quality of life/participation.

First, regarding the effect size and heterogeneity, the pooled effect size (Hedges’ g ≈ 0.19) was small, consistent with the proposed physiological mechanisms of vibration therapy—muscle spindle and Ia afferent activation leading to enhanced muscle activation, sensory feedback, and postural control [45]. However, the fact that the effect became non-significant after publication bias adjustment suggests potential overrepresentation of positive findings in the literature [46]. The moderate heterogeneity (I^2^ ≈ 34%) likely reflects differences in study design, participant stage (acute, subacute, or chronic), intervention parameters (frequency, amplitude, posture, and duration), and outcome tools (basic, instrumental, or quality-of-life ADL measures). For example, studies conducted in the subacute phase (e.g., Lee et al., 2017 [36]; Hwang, 2018 [43]; Kim & Lee, 2021 [39]) generally reported improvements in balance and lower-limb function, whereas chronic-stage trials (e.g., Liao et al., 2016 [34]; Costantino et al., 2017 [35]; Annino et al., 2019 [37]) demonstrated more localized effects or inconsistent ADL gains. This variation likely contributed to the moderate heterogeneity observed in the pooled analysis. Thus, rather than generalizing the effects of vibration therapy to all patients, careful interpretation that considers patient characteristics and parameter suitability is required [47,48]. These findings are consistent with previous evidence: Zeng et al. (2024) reported that whole-body vibration (WBV) improves spasticity, motor, and balance outcomes but showed inconsistent effects on gait, ADL, and quality of life [16]. Park et al. (2018) also found benefits for balance and gait, but evidence for ADL was limited and highly variable [49]. Similarly, Lu et al. (2024) showed upper-limb functional gains with vibration therapy, while disability and independence measures such as FIM or BI remained inconclusive due to heterogeneity in study design and intervention parameters [50]. Some individual RCTs demonstrated positive signals for balance and ADL following six weeks of WBV, but small sample sizes and design variability limited generalizability [32,51]. In summary, our finding of a small effect size, publication bias, and moderate heterogeneity aligns with prior research, suggesting that vibration therapy may provide consistent benefits in physical function, whereas its effects on ADL and quality of life remain uncertain.

Second, regarding the moderating role of session number, meta-regression identified the number of sessions as the only significant moderator. The greatest improvements were observed in the 13–24 session range, while ≤12 sessions produced negligible effects and ≥25 sessions showed diminished benefits [32,50]. This pattern suggests a non-linear dose–response curve, rather than a simple “more is better” relationship [52]. Physiologically, vibration therapy may initially facilitate motor-sensory activation (muscle activation, proprioceptive input, postural strategies), but beyond a threshold, fatigue, habituation, or compensatory rigidity may lead to diminishing or plateau effects. The concept of an optimal dose window has been repeatedly reported in domains beyond ADL, such as balance, gait, and muscle strength, and our results support its importance in ADL outcomes as well. Minimal clinically important difference (MCID) values for the Barthel Index and Functional Independence Measure have been proposed, but between-study heterogeneity in scale version and recovery phase precluded consistent application. Therefore, standardized effect sizes were used for comparability. Clinically, a practical prescription window of approximately 3–6 weeks with 3–5 sessions per week (13–24 sessions in total) may be considered, with individual adjustments based on patient response (e.g., fatigue, dizziness, pain, balance reactions) and concurrent rehabilitation modalities (physical or occupational therapy). This interpretation is consistent with broader stroke motor-rehabilitation guidelines recommending 3–5 therapy sessions per week over 8–12 weeks, corresponding to multi-week, repeated training schedules [53,54].

Notably, our explanatory model showed that session number accounted for about 24% of heterogeneity. In contrast, session duration, weekly frequency, vibration type (WBV vs. FMV), frequency category, and stroke stage were not significant moderators. The amplitude subgroup analysis appeared to fully explain heterogeneity, but this likely reflected model instability and overfitting due to a sharp reduction in sample size. Therefore, while session number optimization can be considered a robust and reliable prescription factor, other parameters (frequency, amplitude, session length, weekly frequency) still lack sufficient evidence and warrant further high-quality trials. The wide variability in vibration parameters across included studies—particularly in frequency (20–40 Hz), amplitude (1–5 mm), posture, and exposure duration—likely contributed to the observed heterogeneity and inconsistent functional outcomes. Although these parameters were not identified as significant moderators in the present meta-regression, the small number of studies reporting standardized details limits interpretability. Future randomized controlled trials should systematically manipulate and report vibration parameters using standardized reporting frameworks to establish reproducible dose–response relationships. In particular, factorial designs that explore frequency–amplitude and time–dose interactions could help define optimal therapeutic combinations for specific functional outcomes, including ADL.

Third, regarding implications for ADL subdomains, significant improvements were observed only in the physical function/mobility domain, but not in self-care/daily activities or quality of life/participation. This suggests that the primary therapeutic target of vibration therapy is sensorimotor and biomechanical function (e.g., trunk control, mobility, gait-related thresholds and strategies), while higher-order outcomes such as independence and quality of life are mediated by multiple factors—including cognitive, emotional, environmental, and social support—that may not respond to short-term physiological interventions [55]. Accordingly, clinical protocols should prioritize mobility, trunk stability, and gait performance as primary outcomes, with ADL independence and quality of life addressed through long-term follow-up and multimodal interventions (e.g., occupational therapy, home environment modification, cognitive or communication rehabilitation) [56]. In this context, vibration therapy—which primarily targets body function and sensorimotor control—should be integrated with occupation-based or task-specific interventions to facilitate the transfer of motor improvements to higher-level ADL outcomes. Combining VT with conventional occupational or physical therapy may bridge the gap between impairment-level gains and meaningful participation in daily life.

Beyond the behavioral findings, the therapeutic effects of VT may be explained by its neurobiological and neurophysiological mechanisms. Peripheral vibration stimulates muscle-spindle Ia-afferents, increasing spinal excitability via the tonic vibration reflex and ascending sensory input through the dorsal column–medial lemniscus pathway to the thalamus and sensorimotor cortex (S1/M1). These inputs are modulated through basal-ganglia–thalamic–cortical and cerebellar–thalamic–cortical circuits that support error-based learning, proprioceptive recalibration, and postural adaptation [57,58]. The cerebellum plays a pivotal role in internal-model updating, inhibitory control, and predictive timing, all essential for coordinated movement. Moreover, the mirror-neuron system (MNS)—including the inferior frontal gyrus and inferior parietal lobule—interacts closely with the cerebellum and may enhance sensorimotor integration and action understanding, thereby facilitating functional recovery during daily activities [59].

Consistent with our findings, which showed that most included studies employed whole-body vibration (WBV) and demonstrated significant improvements in ADL performance, the neurophysiological mechanisms of WBV appear well-suited to promote global functional recovery. WBV delivers a low-amplitude, whole-body mechanical oscillation that stimulates multiple muscle groups simultaneously through postural adjustments and global proprioceptive activation. This mechanism engages spinal reflex loops, vestibulo-spinal pathways, and sensorimotor cortical reweighting, thereby enhancing balance, postural control, and overall motor coordination—key prerequisites for independence in ADL. In contrast, focal muscle vibration (FMV) applies localized, high-frequency mechanical stimulation directly to target muscles or tendons, inducing a tonic vibration reflex and selective Ia-afferent activation, which modulate corticospinal excitability and motor-unit recruitment at the segmental level. FMV thus primarily influences fine motor control, spasticity modulation, and muscle-specific reeducation through focal proprioceptive feedback. However, in the present meta-analysis, the number of FMV studies was limited, and their reported outcomes were insufficient to demonstrate a significant pooled effect on ADL. This scarcity of evidence may reflect the narrower therapeutic scope of FMV, which targets localized motor units rather than whole-body coordination. From a clinical perspective, WBV appears more suitable for patients with global postural or balance deficits, whereas FMV may serve as a complementary intervention for those with focal impairments, such as upper-limb weakness, hand dexterity loss, or persistent spasticity. Further research with adequately powered trials is needed to clarify the distinct roles and potential synergy between these two modalities in post-stroke rehabilitation.

Recent multimodal studies integrating EEG and kinematic analyses have objectively demonstrated VT-induced modulation of cortical oscillations and motor performance, indicating that such parameters may serve as biomarkers of neural reorganization after stroke [60]. Clinically, these findings suggest that VT operates through both bottom-up proprioceptive stimulation and top-down cortical modulation, bridging spinal reflex enhancement and cortical plasticity. Accordingly, future studies should include neurophysiological outcomes such as EEG event-related desynchronization (ERD/ERS), cortico-muscular coherence, and kinematic measures of movement quality and balance to further elucidate the mechanisms underpinning ADL improvement. Furthermore, integrating VT with virtual reality (VR) or non-invasive brain stimulation (NIBS) may synergistically enhance sensorimotor network reorganization and accelerate functional recovery [61].

Limitations of this study must be acknowledged. First, publication bias was detected, and sensitivity analyses demonstrated loss of significance, lowering confidence in the conclusions. Second, methodological quality varied, with some RCTs rated as high or unclear risk for randomization, allocation concealment, blinding, or ITT analysis, necessitating cautious interpretation. Third, ADL assessment tools varied widely (basic, instrumental, quality of life), and reliance on estimated change scores or digitized data may have introduced error. Fourth, intervention parameters were heterogeneous (frequency, amplitude, posture, set–rest configurations), limiting dose–response mapping. Fifth, the subgroup analysis by amplitude should be interpreted with caution, as the limited number of studies in each subgroup may have caused model instability or overfitting; therefore, these findings are exploratory rather than confirmatory. Finally, the small sample sizes and relatively short intervention or follow-up durations across included trials restricted the ability to evaluate long-term or cumulative effects.

Future research should aim to (i) confirm the optimal session window of 13–24 sessions in prospective dose–response RCTs; (ii) systematically test frequency, amplitude, posture, and set–rest combinations using factorial designs to delineate non-linear dose–response curves; (iii) establish a core outcome set (COS) to standardize concurrent and longitudinal assessment of ADL and physical function; and (iv) incorporate patient-level moderators (stroke stage, baseline balance/sensory deficits, cognition, assistive device use) to develop and validate personalized prescription algorithms.

## 5. Conclusions

Vibration therapy demonstrated a small but statistically significant effect on ADL in stroke patients; however, this effect became uncertain after adjustment for publication bias. Nevertheless, session number emerged as a significant moderator, explaining approximately 24% of heterogeneity, with the 13–24 session range representing a clinically realistic optimal dose window. Given that improvements are more likely to occur initially in physical function and mobility rather than in overall ADL independence, clinical protocols should prioritize motor recovery as a primary goal, while targeting ADL and quality-of-life improvements through multimodal interventions and long-term follow-up. At the same time, evidence for parameters other than session number remains insufficient, highlighting the need for standardized dose–response investigations and high-quality large-scale randomized controlled trials.

## Figures and Tables

**Table 1:** Study characteristics of 13 studies selected for the meta-analysis.

Author & Year	Study Type	Participants	Sample (N, Age)	Intervention & Control Protocol	Intervention Protocol	WBV Parameters	Posture or Irradiation Site	Device Type	Outcome Measures
van Nes et al. (2006) [32]	RCT	Subacute	I: 27 (59.7 ± 12.3) C: 26 (62.6 ± 7.6)	I: WBV + Ex C: Ex	Freq: 5 sess./wk, Dur: 6 wk	Bouts: 4, Bout Dur: 0.75 min, Rest: 1 min between bouts, Freq: 30 Hz, Amp: 3 mm	Squatting	Galileo 900 (Galileo Therapy, Pforzheim, Germany)	BI, TCT, RMI
Merkert et al. (2011) [33]	RCT	Subacute	I: 25 (74.5 ± 8.3) C: 23 (74.5 ± 8.6)	I: WBV + Ex C: Ex	Freq: 5 sess./wk, Dur: 3 wk	Bouts: 6, Bout Dur: 0.5 min, Freq: 35 Hz	Sitting	Vibrosphere (ProMedVi, Lund, Sweden)	BI
Liao et al. (2016) [34]	RCT	Chronic	I1: 28 (60.8 ± 8.3) I2: 28 (62.9 ± 10.2) C: 28 (59.8 ± 9.1)	I1: WBV (20 Hz) + Ex I2: WBV (30 Hz) + Ex C: Ex	Freq: 6 sess./wk, Dur: 4 wk	Bouts: 4, Bout Dur: 1.5 min, Rest: 1.5 min between bouts, Freq: 20 or 30 Hz, Amp: 1 mm	Squatting	Fitvibe Pro (Gymna, Diepenbeek, Belgium)	FAI, CHIEF-C, SF-12-Physical, PCS, MCS
Costantino et al. (2017) [35]	RCT	Chronic	I: 17 (62.59 ± 15.39) C: 15 (60.47 ± 16.09)	I: FMV + Ex C: Sham	Freq: 3 sess./wk, Dur: 4 wk	Bouts: 1, Bout Dur: 30 min, Freq: 300 Hz, Amp: 2 mm	Triceps + Extensor carpi radialis	Viss One Evolution (Vissman, Pescantina, Italy)	QDS, FIM
Lee et al. (2017) [36]	RCT	Subacute	I: 15 (59.1 ± 16.9) C: 15 (64.4 ± 14.8)	I: WBV + Ex C: Ex	Freq: 5 sess./wk, Dur: 2 wk	Bouts: 1, Bout Dur: 30 min, Freq: 40 Hz	Sitting	Sonix^®^ (Sonic World, Wonju, Republic of Korea)	K-MBI, FAC, TIS-T, TIS-S, TIS-D, TIS-C
Annino et al. (2019) [37]	RCT	Chronic	I: 17 (67.8 ± 8.3) C: 17 (69.4 ± 10.4)	I: FMV + Ex C: Ex	Freq: 3 sess./wk, Dur: 8 wk	Bouts: 1, Bout Dur: 5 min, Freq: 30 Hz, Amp: 2 mm	Triceps of the hemiparetic arm	Segmental vibration device (self-development)	BI
Wei et al. (2019) [38]	RCT	Subacute	I: 32 (59.19 ± 11.25) C1: 25 (60.44 ± 10.38) C2: 22 (60.14 ± 9.79)	I: FMV C1: Sham C2: TAU	Freq: 7 sess./wk, Dur: 4 wk	Bouts: 18, Bout Dur: 0.083 min, Rest: 10 min between bouts, Freq: 196 Hz, Amp: 0.3 mm	Wrist	SCW-V2 (HK Polytecnic Univ., Hong Kong)	FIM
Kim et al. (2021) [39]	RCT	Subacute	I: 20 (57.20 ± 11.00) C: 18 (55.70 ± 10.40)	I: WBV + Ex C: Ex	Freq: 10 sess./wk, Dur: 2 wk	Bouts: 1, Bout Dur: 20 min, Freq: 16 Hz	Squating	Sonix SW-VM10 (Sonic World, Wonju, Republic of Korea)	FAC
Yoon et al. (2023) [40]	Non-RCT	Subacute	I: 22 (64.68 ± 8.82) C: 22 (67.04 ± 8.85)	I: WBV + Ex C: Ex	Freq: 5 sess./wk, Dur: 8 wk	Bouts: 1, Bout Dur: 30 min, Freq: 20–30 Hz	Standing	Galileo 900 (Galileo Therapy, Pforzheim, Germany)	TIS
Majeed et al. (2024) [41]	RCT	Chronic	I: 16 (63.87 ± 6.87) C: 16 (63.37 ± 7.94)	I: WBV + Ex C: Ex	Freq: 3 sess./wk, Dur: 6 wk	Bouts: 1, Bout Dur: 5 min, Freq: 20–40 Hz	Sitting	OTO electro reflexologist ER-839S (OTO Bodycare, Singapore)	SS-QOL. MPASS
Annino et al. (2025) [42]	RCT	Chronic	I: 19 (67.8 ± 8.3) C: 18 (69.4 ± 10.4)	I: FMV + Ex C: Ex	Freq: 3 sess./wk, Dur: 8 wk	Bouts: 3, Bout Dur: 10 min, Rest: 1 min between bouts, Freq: 30 Hz, Amp: 0.2 mm	Quadriceps tendon	FOV1 Power Club (Ferrara, Italy)	BI
Hwang et al. (2018) [43]	RCT	Subacute	I: 9 (56.0–76.0) C: 9 (66.0–73.0)	I: WBV + Ex C: Ex	Freq: 5 sess./wk, Dur: 4 wk	Bouts: 1, Bout Dur: 10 min, Freq: 20–30 Hz, Amp: 2–3 mm	Standing	Galileo Med S (Novotec Medical GmbH, Pforzheim, German)	FAC, MBI-T, MBI-S, MBI-M
Alp et al. (2018) [44]	RCT	Chronic	I: 10 (61.2 ± 11.0) C: 11 (62.9 ± 8.2)	I: WBV + Ex C: Ex	Freq: 3 sess./wk, Dur: 4 wk	Bouts: 1, Bout Dur: 5 min, Freq: 40 Hz, Amp: 4 mm	Standing	Compex Winplate (Compex, Vista, CA, USA)	FIM

Abbreviations. Amp, amplitude; BI, Barthel Index; Bouts, vibration bouts (sessions within a treatment); C, control group; CHIEF-C, Craig Hospital Inventory of Environmental Factors—Chinese version; Dur, duration; Ex, exercise; FAC, functional ambulation categories; FAI, Frenchay Activity Index; FIM, Functional Independence Measure; Freq, frequency; FMV, focal muscle vibration; I, intervention group; K-MBI, Korean version of the Modified Barthel Index; MBI-Mobility, Modified Barthel Index—Mobility subscale; MBI-Self-care, Modified Barthel Index—Self-care subscale; MBI-Total, Modified Barthel Index—Total score; MCS, mental component summary of SF-12; N, sample size; PCS, physical component summary of SF-12; QDS, Quick Disabilities of the Arm, Shoulder and Hand Questionnaire (QuickDASH); RCT, randomized controlled trial; RMI, Rivermead Mobility Index; Sess., sessions; SF-12, 12-Item Short Form Health Survey; SS-QOL, Stroke-Specific Quality of Life; TAU, treat as usual; TCT, trunk control test; TIS-C, Trunk Impairment Scale—Coordination subscale; TIS-D, Trunk Impairment Scale—Dynamic sitting balance subscale; TIS-S, Trunk Impairment Scale—Static sitting balance subscale; TIS-T, Trunk Impairment Scale—Total score; WBV, whole-body vibration; Wk, week.

**Table 2:** Results of meta-regression analyses for moderator variables of WBV effects on ADL.

Moderator Variable	Level	Reference Level	β	95% CI	*t*	*p*-Value	F (df_1_, df_2_)	*p*
Total session number	13–24	≥25	0.34	[0.048, 0.632]	2.37	0.024 *	3.408 (2, 34)	0.045 *
	≤12		0.005	[−0.356, 0.367]	0.03	0.976		
Duration per 1 session	≤5 min	6–20 min	0.125	[−0.233, 0.483]	0.71	0.483	0.857 (2, 32)	0.434
	≥30 min		−0.136	[−0.504, 0.232]	−0.752	0.458		
Stroke type	chronic	subacute	−0.034	[−0.319, 0.250]	−0.246	0.807	0.061 (1, 35)	0.807
Amplitude type	≤1 mm	>1 mm	−0.133	[−0.363, 0.097]	−1.196	0.245	1.430 (1, 22)	0.245
Vibration type	FMV	WBV	−0.043	[−0.422, 0.336]	−0.230	0.82	0.053 (1, 35)	0.82
Frequency type	31–100 Hz	21–30 Hz	0.181	[−0.171, 0.533]	1.045	0.303	0.378 (3, 33)	0.769
	≤20 Hz		0.036	[−0.359, 0.432]	0.188	0.852		
	≥101 Hz		0.072	[−0.407, 0.550]	0.305	0.762		

β = meta-regression coefficient (difference in effect size relative to the reference level); CI = 95% confidence interval; df = degrees of freedom; F (df_1_, df_2_) = test of moderators. Fixed effects tested using Knapp and Hartung adjustment. *p*  <  0.05 is considered statistically significant and is indicated with an asterisk (*).

**Table 3:** Heterogeneity statistics and explained variance across moderator models.

Model	Q(df)	*p*	τ^2^	I^2^ (%)	R^2^ Analog (%)
Baseline (no moderators)	Q(36) = 56.90	0.015	0.052	33.9	–
Stroke type (univariate)	Q(35) = 56.79	0.011	0.058	36.1	0
Vibration type (univariate)	Q(35) = 56.87	0.011	0.058	36.3	0
Total Session number (univariate)	Q(34) = 48.85	0.048	0.044	30.2	24
Duration per 1 session (univariate)	Q(32) = 54.13	0.009	0.071	40.1	0
Frequency type (univariate)	Q(33) = 54.11	0.012	0.058	35.8	0
Amplitude type (univariate)	Q(22) = 15.66	0.832	0	0	100

## Data Availability

The data supporting the findings of this study are available from the corresponding author upon reasonable request.

## Supplementary Materials

The following supporting information can be downloaded at https://www.mdpi.com/article/10.3390/jcm14217682/s1, Table S1: PRISMA Checklist; Text S1: Search strategies.
