# Supplementary material for: The Effects of Vibration Therapy on Activities of Daily Living After Stroke: A Systematic Review and Meta-Analysis
Source: J Clin Med. 2025 Oct 29;14(21):7682. doi: 10.3390/jcm14217682 (PMC12608700; doi:10.3390/jcm14217682)
Supplement: Supplementary file 1 [file jcm-14-07682-s001.zip › jcm-3939927- Text S1.pdf]

Supplementary Text S1. Search Strategies

The complete draft search strategies for all databases used in this review are presented below. Boolean operators, field tags, and filters are specified to ensure reproducibility.

PubMed

(“vibration therapy”[Title/Abstract] OR “whole body vibration”[Title/Abstract] OR “WBV”[Title/Abstract] OR “focal muscle vibration”[Title/Abstract] OR “FMV”[Title/Abstract] OR “segmental vibration”[Title/Abstract] OR “vibration intervention”[Title/Abstract] OR “vibration”[Title/Abstract])

AND

(“activities of daily living”[Title/Abstract] OR “ADL”[Title/Abstract] OR “functional independence”[Title/Abstract] OR “Barthel Index”[Title/Abstract] OR “FIM”[Title/Abstract] OR “Frenchay Activities Index”[Title/Abstract] OR “SF-12”[Title/Abstract] OR “stroke-specific quality of life”[Title/Abstract])

AND

(“stroke”[Title/Abstract] OR “post-stroke”[Title/Abstract] OR “cerebrovascular accident”[Title/Abstract])

AND

(“randomized controlled trial”[Publication Type] OR “controlled clinical trial”[Publication Type] OR randomized[Title/Abstract] OR randomly[Title/Abstract] OR trial[Title/Abstract])

Embase

(‘stroke’/exp OR stroke*:ti,ab OR “cerebrovascular accident”:ti,ab OR “post-stroke”:ti,ab)

AND

(‘vibration therapy’/exp OR “whole body vibration”:ti,ab OR “WBV”:ti,ab OR “focal muscle vibration”:ti,ab OR “FMV”:ti,ab OR “segmental vibration”:ti,ab OR “vibration”:ti,ab)

AND

(‘activities of daily living’/exp OR ADL:ti,ab OR “functional independence”:ti,ab OR “Barthel Index”:ti,ab OR FIM:ti,ab OR “Frenchay Activities Index”:ti,ab OR “SF-12”:ti,ab OR “stroke-specific quality of life”:ti,ab)

AND

(‘randomized controlled trial’/exp OR random*:ti,ab OR placebo:ti,ab OR trial:ti,ab)

Web of Science

TS=(stroke* OR “post-stroke” OR “cerebrovascular accident”)

AND

TS=(“vibration therapy” OR “whole body vibration” OR “WBV” OR “focal muscle vibration” OR “FMV” OR “segmental vibration” OR “vibration intervention” OR vibration)

AND

TS=(“activities of daily living” OR “functional independence” OR ADL OR “Barthel Index” OR “FIM” OR “Frenchay Activities Index” OR “stroke-specific quality of life” OR “SF-12”)

AND

TS=(random* OR trial*)

Cochrane Library

(MeSH descriptor: [Stroke] explode all trees OR stroke:ti,ab,kw OR “cerebrovascular accident”:ti,ab,kw)

AND

(“vibration therapy”:ti,ab,kw OR “whole body vibration”:ti,ab,kw OR “WBV”:ti,ab,kw OR “focal muscle vibration”:ti,ab,kw OR “FMV”:ti,ab,kw OR “segmental vibration”:ti,ab,kw OR “vibration intervention”:ti,ab,kw OR vibration:ti,ab,kw)

AND

(“activities of daily living”:ti,ab,kw OR “ADL”:ti,ab,kw OR “functional independence”:ti,ab,kw OR “Barthel Index”:ti,ab,kw OR FIM:ti,ab,kw OR “Frenchay Activities Index”:ti,ab,kw OR “stroke-specific quality of life”:ti,ab,kw)

AND

(random*:ti,ab,kw OR “controlled clinical trial”:pt OR “randomized controlled trial”:pt)

Scopus

TITLE-ABS-KEY(stroke* OR “post-stroke” OR “cerebrovascular accident”)

AND

TITLE-ABS-KEY(“vibration therapy” OR “whole body vibration” OR “WBV” OR “focal muscle vibration” OR “FMV” OR “segmental vibration” OR “vibration intervention” OR vibration)

AND

TITLE-ABS-KEY(“activities of daily living” OR ADL OR “functional independence” OR “Barthel Index” OR FIM OR “Frenchay Activities Index” OR “SF-12” OR “stroke-specific quality of life”)

AND

TITLE-ABS-KEY(random* OR trial*)
